# Can psychosocial risk factors mediate the association between precarious employment and mental health problems in Sweden? Results from a register-based study

**DOI:** 10.5271/sjweh.4151

**Published:** 2024-05-01

**Authors:** Fabrizio Méndez-Rivero, Nuria Matilla-Santander, Virginia Gunn, David H Wegman, Julio C Hernando-Rodriguez, Signild Kvart, Mireia Julià, Bertina Kreshpaj, Theo Bodin, Tomas Hemmingsson, Carles Muntaner, Eva Padrosa, Melody Almroth

**Affiliations:** 1 GREDS (Research Group on Health Inequalities, Environment, and Employment Conditions Network), Universitat Pompeu Fabra, Barcelona, Spain.; 2 Unit of Occupational Medicine, Institute of Environmental Medicine, Karolinska Institutet, Stockholm, Sweden.; 3 MAP Centre for Urban Health Solutions, Li Ka Shing Knowledge Institute, Unity Health Toronto, Toronto, Canada.; 4 School of Nursing, Cape Breton University, Nova Scotia, Canada.; 5 University of Massachusetts Lowell, Lowell, USA,; 6 ESIMar (Mar Nursing School), Parc de Salut Mar, Universitat Pompeu Fabra-affiliated, Barcelona, Spain.; 7 SDHEd (Social Determinants and Health Education Research Group), IMIM (Hospital del Mar Medical Research Institute), Barcelona, Spain.; 8 Section of Epidemiology, Department of Public Health, University of Copenhagen, Copenhagen, Denmark.; 9 Centre for Occupational and Environmental Medicine, Stockholm Region, Stockholm, Sweden.; 10 Institute of Environmental Medicine, Karolinska Institutet, Stockholm, Sweden.; 11 Centre for Social Research on Alcohol and Drugs, Stockholm University, Stockholm, Sweden.; 12 Lawrence S. Bloomberg Faculty of Nursing, St. George Campus, Toronto, Canada.; 13 Dalla Lana School of Public Health, University of Toronto, Toronto, Canada.; 14 Department of Mental Health, The Johns Hopkins University Bloomberg School of Public Health, Baltimore, USA.

**Keywords:** mental health, mediation analysis, psychological work environment

## Abstract

**Objectives:**

The aim of this study was to examine the mediating effect of the psychosocial work environment on the association between precarious employment (PE) and increased risk of common mental disorders (CMD), substance use disorders and suicide attempts.

**Methods:**

This longitudinal register-study was based on the working population of Sweden, aged 25–60 years in 2005 (N=2 552 589). Mediation analyses based on a decomposition of counterfactual effects were used to estimate the indirect effect of psychosocial risk factors (PRF) (mediators, measured in 2005) on the association between PE (exposure, measured in 2005) and the first diagnosis of CMD, substance use disorders, and suicide attempts occurring over 2006–2017.

**Results:**

The decomposition of effects showed that the indirect effect of the PRF is practically null for the three outcomes considered, among both sexes. PE increased the odds of being diagnosed with CMD, substance use disorders, and suicide attempts, among both men and women. After adjusting for PE, low job control increased the odds of all three outcomes among both sexes, while high job demands decreased the odds of CMD among women. High job strain increased the odds of CMD and suicide attempts among men, while passive job increased the odds of all three outcomes among women.

With no internationally standardized definition, precarious employment (PE) remains undefined. Recently, however, the literature has tended to converge towards a common notion that refers to a generalized phenomenon of employment insecurity, income inadequacy, and a lack of rights and protection, which has globally grown in recent decades (1, 2), even in countries such as Sweden with a long-standing tradition of protected labor relations (3).

According to this notion, PE is defined in terms of employment rather than working conditions. The terms “employment conditions” and “working conditions” represent distinct yet interconnected concepts. The former are established through contracts between employers and workers, outlining aspects such as wage payments, working conditions, and the extent of social protection afforded to employees. On the other hand, working conditions encompass the specifics of tasks undertaken by workers, encompassing elements like the physical and chemical environment, ergonomic conditions, psychosocial factors, and technological utilization. Additionally, working conditions encompass hierarchical structures, power dynamics, employee involvement in decision-making processes, and issues related to workplace discrimination (4). Thus, although PE is expressed in both employment and working conditions, its fundamental causes are to be found in the flexibilization of the labor market, the deregulation of labor relations and the lack of social protection for workers (1, 5, 6).

## Psychosocial work environment as a pathway linking precarious employment and mental health

Mental disorders are considered a major public health problem in European countries and often studied on their own (7) or in connection with other factors. For instance, an increasing number of studies examine mental disorders in relation to PE (2, 8).

In spite of these advances, the causal mechanisms that allow the effect of PE on mental health to be conveyed have not been sufficiently explored, especially empirically (8, 9). Some conceptual frameworks postulate that the psychosocial work environment could be an intermediate step in a causal pathway that links economic, social, cultural, and political structures with health and disease through psychological and psychophysiological processes (1, 10, 11).

The demand–control model is a widely used framework to assess the influence of the psychosocial work environment on health. This model defines high strain jobs as the result of a combination of high job demands and low job control (poor job decision latitude) (12, 13). For instance, four reviews providing good quality evidence in support of a strong relationship between high strain and clinical depression were included in a recent systematic review with meta-analysis (14). Another systematic meta-review found a moderate quality level of evidence, from multiple prospective studies, that high demands and low control, among other psychosocial risk factors (PRF), are associated with a greater risk of developing common mental health problems (15). (14) for Sweden, two recent longitudinal studies have shown that on the one hand, lower control was associated with an increased risk of depression, whilst higher demands tended to be associated with a slight decrease in risk for depression, among men and women (with differences between sexes) (16). On the other hand, high strain and passive jobs (both low control jobs) were associated with an increased risk of depression among men, and passive jobs were associated with an increased risk among women (17).

Further, recent work has demonstrated the value of using a job exposure matrix (JEM) approach in epidemiological studies by measuring the psychosocial working conditions independently of the health outcome, thus, establishing the level of psychosocial exposure of different occupations from survey data (16, 18, 19). The JEM score can be easily assigned to individuals based on their occupations, replacing the need for time consuming case-by-case assessments. Also, JEM allow occupations to be linked to exposures in a systematic manner and, if a misclassification bias occurs, it is expected that this is non-differential with respect to health (20).

Additionally, recent cross-sectional studies provide new evidence about the relationship between the psychosocial work environment and PE. One such study showed that precarious workers from Stockholm County are more likely to experience psychosocial hazards, and these experiences are more prevalent among women than men (21). Another study based on data from 23 European countries found that the association of PE with poor mental health was completely attributable to the PE–PRF interaction among women, and partially attributable among men (22). However, both studies are based on cross-sectional data; therefore, they do not allow measuring the effect of PE and PRF on mental health over time.

## Gender differences

Previous research has also identified that gender may interact with some dimensions of PE, creating differential effects on the health of men and women (23, 24). Besides, other results suggest that gender inequalities in exposure to PRF at work have more detrimental effects on women’s mental health (25). However, there are very few studies examining gender differences in the effect of multidimensional PE on mental health, as well as the role of PRF in this relationship (26).

Building on this theoretical and empirical background, we hypothesized that PRF mediate the association between PE and workers’ mental health. Accordingly, the main objective of this study is to analyze the potential mediating effect of factors such as 'low control', 'high demands', 'high strain', and 'passive job' on the PE–poor mental health relationship among workers in Sweden, with consideration for differences between women and men.

## Methods

The present study is based on the Swedish Work, Illness, and Labor-market Participation (SWIP) cohort, which contains linked information from multiple registers and includes all registered individuals in Sweden, aged 16–65 years (approximately 5.4 million) in 2005 and followed until the end of 2016 (20). After exclusions, this study uses a subpopulation of 2 552 589 individuals aged 25–60 years old who were residing in Sweden in 2005. Exclusion criteria for the study were defined as: (i) incomplete information for measuring the exposure variable (2005), mediators (2005) and outcomes (2006–2016) (401 167 excluded); (ii) individuals who emigrated or died during follow up (113 773 excluded); (iii) individuals with income lower than 100SEK in 2005 – considered unemployed (625 178 excluded); (iv) self-employed in 2005 as self-employed individuals may be classified as precariously employed when this is not necessarily the case (301 081 excluded); (v) unemployed for >6 months in 2005, in order to differentiate the effects of long-term unemployment situations from PE (88 011 excluded); (vi) students in 2005 (176 349 excluded); and (vii) individuals receiving pension benefits or early pension benefits in 2005 to avoid misclassifying individuals leaving the labor market into PE (91 340 excluded).

### Outcomes

Workers’ mental health was measured through diagnosed CMD (depression, anxiety, and stress-related disorders; ICD-10 codes F32, F33, F41, F43), and alcohol and substance use disorders (ICD-10 codes F10-F19) reported in the in- and outpatient registers during the follow-up period (2006–2016). Like previous studies, only suicide attempts (ICD-10 codes X60-X84 and Y10-Y34) reported in the inpatient register were included (27, 28). Both registers are national and comprehensive of the Swedish population. The validity of the inpatient register is high despite some limitations for psychiatric diagnoses (29).

### Exposure

PE was measured through the Swedish Register based Operationalization of Precarious Employment (SWE-ROPE), a five-item multidimensional measurement that encompasses three dimensions, namely, employment insecurity, income inadequacy, and lack of rights and protection (30). A PE score was computed as the sum of all five items, ranging from -9 to +2, with -9 being the most precarious. The PE score was then transformed into a dichotomous variable (PE score <-3 = PE; PE score ≥ -3 = non-PE). The cut-off point (-3) was defined based on a recent analysis using the same data (31).

### Mediators

In the present study, the PRF were addressed from the demand–control model, and measured through the Swedish psychosocial JEM, which was developed using data from the Swedish Work Environment Surveys from 1997 to 2013 and averaged for each occupation (around 350 occupations). For this analysis, these scores were linked to the individuals in the study population based on their registered occupation in 2005 (16). Next, demand and control variables were dichotomized using the median as the cut-off point (16).

Finally, the job demands indicator was plotted against job control and four groups were created: high control/high demands (active jobs), high control/low demands (low strain jobs), low control/high demands (high strain jobs), and low control/low demands (passive jobs) (32). In addition to low control and high demands, high strain and passive jobs are also widely recognized as PRF (33). In the supplementary material (www.sjweh.fi/article/4151), table S1 [(from (16)] shows the English translation of items used for assessing control and demands. Information on the construction of JEM is extensively described elsewhere, including information on validity and reliability (34).

### Covariates

**Figure 1:**
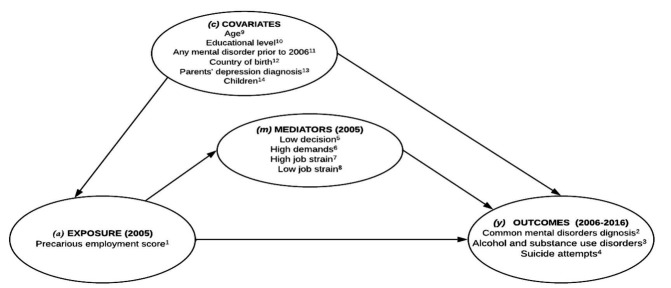
Diagram of the total and mediated effect. ^1^ Binary, 1=precarious; ^2^ Binary, 1=reported in the inpatient and outpatient registers; ^3^ Binary, 1=reported in the inpatient and outpatient registers; ^4^ Binary, 1=reported in the inpatient registers; ^5^ Binary, 1=low; ^6^ Binary, 1=high; ^7^ Binary, 1=high; ^8^ Binary, 1=passive; ^9^ Age (group of age); ^10^ Educational level (categorical); ^11^ Country of birth (binary, 1=Sweden); ^12^ Any mental disorder prior to 2006 (binary, 1=reported); ^13^ Parents' depression diagnosis (binary, 1=reported); ^14^ Children (binary, 1=at least 1 child).

We draw our causal assumptions in a diagram (figure 1) for identifying the minimal sufficient set of variables for adjustment. The variables were: (i) age, categorized into 25–35, 36–50, and 51–60 years; (ii) educational level, measured based on the number of years of schooling, using three categories 1=≤9 years; 2=10–15 years; 3=>15 years; (iii) country of birth (born in Sweden or not); (iv) children (have ≥1 child or 0); (v) previous psychiatric diagnoses based on information obtained from the inpatient register and defined as having any psychiatric diagnosis (ICD-10 codes F00 to F99) before 2006 (the first year of follow-up) – this adjustment was done to rule out reverse causation; and (vi) parents’ depression diagnoses were obtained by linking the index person to their parents’ inpatient records from 1973 (when the registry began) onward, specifying whether either parent had a first-time depression diagnosis prior to age 65 (16).

### Statistical analysis

For this study, mediation analysis based on a decomposition of effects was conducted to estimate the indirect effect of low control, high demands, high strain, and passive job in 2005 on the association between PE in 2005 and diagnosis of CMD (ie, depression, anxiety and stress-related disorders), alcohol and substance use disorders, and suicide attempts, during the 2006–2016 timeframe. Previous studies based on the SWIP cohort analyzed separately the association between PE and mental health outcomes (a→y, as shown in figure 1), as well as the association between psychosocial risks and mental health outcomes (m→y). This is the first study to analyse the effect of psychosocial risks on the association between PE and mental health


m↗      ↘ a   →    y .

Exposure and mediators were measured for the same year based on the premise that psychosocial risks are inherent to PE and, although theoretically there is a causal relationship between them, a temporal sequence cannot be established. The user-written Stata command ‘paramed’ extends the traditional Baron and Kenny mediation procedure (35) to allow for the presence of exposure–mediator interactions in the outcome regression and uses counterfactual definitions of direct and indirect effects. In a counterfactual framework, the individual causal effect of the exposure on the outcome is defined as the hypothetical contrast between the outcomes that would be observed in the same individual at the same time in the presence of the exposure and in the absence or in the presence of the exposure of two different levels of the exposure (36).

Two logistic regression models were estimated: one for the mediators’ conditional (adjusted) on exposure and specified covariates and the second for the outcomes conditional on exposure, the mediators, and the same covariates.

The paramed command only supports exposures and mediators that are continuous or dichotomous, it does not support categorical variables with more than two categories. Furthermore, two values must be specified for the exposure: the base (a1) and alternative (a2) level. For the mediator, it is necessary to specify a value ‘m’ representing the level at which the controlled direct effect is to be estimated (37). Although the PE score, as well as control and demands, are available as continuous variables, we do not have any theoretical, statistical, or empirical criteria to define a priori values for (a1), (a2) and (m). For this reason, and given the impossibility of using multi-categorical variables, we have chosen to define exposure and mediators as binary variables.

According to Valeri & Vanderweele (38), the controlled direct effect indicates the effect of being exposed to PE on the risk of suffering the mental outcome if the mediator (PRF) were controlled uniformly at a fixed value. The natural indirect effect expresses the proportion of the effect of the exposure on the outcome attributable to the mediator (38). Natural indirect effect is the most critical component of the effect decomposition because it is the one that enables testing of the mediation hypothesis. For this study, the natural indirect effect of the PRF made it possible to assess their possible mediating role in the association of PE with mental health outcomes.

Mediation analysis is a widely used approach in studies that rely on the need to disentangle the different pathways that could explain the effect of an exposure on an outcome. Typically, this method enables the decomposition of the effect of an exposure on an outcome (total effect) into two components: the effect of the exposure that is explained by the mediators (indirect effect) and the effect of the exposure unexplained by those same mediators (direct effect) (36).

In addition, five sensitivity analysis were performed. First, individuals with a PE score of -3– -1 were excluded. These individuals are at the borderline of PE and could introduce a misclassification diluting the effect estimates so were excluded from the reference group. Supplementary table S2 presents both the controlled direct and natural indirect effect of PE on CMD, alcohol and substance use disorders, and suicide attempt separately for men and women. Second, the robustness of mediator cut-off points was also assessed, using the lowest quartile of job control and the upper quartile of job demands to define low control and high demands, respectively. Third, the mediation analysis was conducted for a shorter follow-up period, from 2006 to 2010, to test the effects closer to the exposure. Fourth, models were adjusted without including previous psychiatric diagnoses as a covariate to address reverse causation and the potential issue regarding healthy worker effect. Fifth, considering that psychosocial risks are measured at the occupational level and PE is measured at the individual level, the variance in psychosocial risks could be underestimated. A multilevel approach was applied, conducting the whole analysis with both psychosocial risks and PE at the occupational level. The proportion of people with a PE score of <-3 was estimated for each occupation and then dichotomized using the median as the cut-off point. Supplementary table S3 presents the multilevel estimation of both the controlled direct and natural indirect effect of PE on CMD, alcohol and substance use disorders, and suicide attempt separately for men and women.

## Results

The sociodemographic and health characteristics of the study sample are presented in table 1. In 2005, 5.17% of men and 6.86% of women were in PE. The proportion of low control and passive job was higher among workers in PE for men and women, while the category of high demands was more frequent among workers not in PE for both sexes. The category of high strain was more frequent only among precariously employed men. Those in PE had a higher proportion of CMD, substance use disorders, and suicide attempts during 2006–2016.

**Table 1:** Characteristics of the study population stratified by precarious employment (PE) status and sex.

		Men						Women				
		Precarious (PE score=0) (N=66 089, 5.17%)			Non-precarious (PE score=1) (N=1 213 186, 94.83%)			Precarious (PE score=0) (N=87 360, 6.86%)			Non-Precarious (PE score=1) N=1 185 954, 93.14%)	
		Frequency	%		Frequency	%		Frequency	%		Frequency	%
Age in 2005 (years)												
	25–35	32 734	49.53		336 167	27.71		43 579	49.88		298 786	25.19
	36–50	23 082	34.93		555 568	45.79		31 869	36.48		543 171	45.8
	51–60	10 100	15.28		318 399	26.24		11 669	13.36		341 208	28.77
Educational level in 2005 (years at school)												
	<9	11 216	16.97		171 954	14.17		10 609	12.14		110 364	9.31
	10–15	43 331	65.56		800 740	66		56 406	64.57		765 093	64.51
	>15	10 980	16.61		238 357	19.65		19 972	22.86		309 362	26.09
Country of birth other than Sweden (ref)		12 248	18.55		113 437	9.35		13 661	15.65		125 622	10.59
≥1 child (ref)		23 047	34.87		578 623	47.69		50 123	57.38		640 249	53.99
Any psychiatric diagnosed prior to 2006 (ref)		3 765	5.7		39 977	3.3		4 024	4.61		40 801	3.44
Depression diagnosis for either parent (ref)		2 087	3.16		32 065	2.64		2 765	3.17		29 401	2.48
Mental health outcomes												
	Common mental disorders reported: in- / outpatient registers (ref)	5473	8.28		57 618	4.75		9 767	11.18		85 610	7.22
	Alcohol & substance use disorders reported: in- / outpatient registers (ref)	3161	4.78		29 736	2.45		1 782	2.04		16 024	1.35
	Suicide attempts reported: inpatient registers (ref)	479	0.72		4 129	0.34		732	0.84		5 300	0.45
Psychosocial risk factors (based on Swedish Job Exposure Matrix)												
	Low control (ref)	42 828	64.8		603 197	49.72		48 616	55.65		587 884	49.57
	High demands (ref)	31 580	47.78		600 181	49.47		29 418	33.67		585 650	49.38
	High strain (ref)	18 838	28.5		184 313	15.19		13 341	15.27		238 501	20.11
	Passive job (ref)	23 990	36.3		418 884	34.53		35 275	40.38		349 383	29.46

The association between PRF and PE expressed as odd ratios (OR) adjusted for covariates was estimated, separately for men and women. In 2005, PE was positively associated with low control (OR 1.53; 95% CI 1.51–1.56), high demands (OR 1.17; 95% CI 1.15–1.19) and high strain (OR 2.21; 95% CI 2.17–2.25), but negatively with passive job (OR 0.84; 95% CI 0.82–0.85), among men. Aversely, among women, PE showed a negative association for high demands (OR 0.55; 95% CI 0.54–0.56) and high strain (OR 0.81; 95% CI 0.79–0.82), and a positive association for low control (OR 1.18; 95% CI 1.17–1.20) and passive job (OR 1.41, 95% CI 1.39–1.43). In addition, the interactions between exposure and mediators were checked. We found significant interactions for men and women. For men, these were 'CMD–high strain', 'substance use disorders–low control', 'substance use disorders–passive job', 'suicide attempt–low control'. For women, CMD interacted with the four mediators: 'substance use disorders–high demands', 'high strain–passive job'; 'suicide attempt–low control', 'high demands–high strain' (data not shown).

Table 2 shows the associations of mental health outcomes with the exposure indicators and mediators, adjusted for covariates and presented separately for men and women. The OR corresponding to PE shows the association between PE and the outcome after adjusting for covariates and the mediators (low control, high demands, high strain and passive job). The OR corresponding to each mediator shows its association with the outcome after adjusting for covariates and PE. Paramed fits one model for each mediator separately, hence, there are four different models for each outcome.

**Table 2:** Odds ratios (OR) for common mental disorders, alcohol and substance use disorders, and suicide attempt (2006–2016) conditional on precarious employment (PE) (in 2005), mediators (in 2005), and adjusted for covariates. [CI=confidence interval; OR=odds ratio: ref=reference.]

				Men				Women		
				Cases/Exposed	OR^a^	95% CI		Cases/Exposed	OR ^a^	95% CI
**Common mental disorders**										
	**Model 1**									
		PE	Non-PE ^b^ (ref)							
			PE	5473/66 089	1.52	1.44–1.60		9767/87 360	1.36	1.31–1.41
		Control	High control ^c^ (ref)							
			Low control	37 649/646 025	1.28	1.26–1.31		51 852/636 500	1.15	1.13–1.16
	**Model 2**									
		PE	Non-PE ^b^ (ref)							
			PE	5473/66 089	1.51	1.45–1.58		9767/87 360	1.31	1.27–1.35
		Demands	Low demands ^d^ (ref)							
			High demands	27 921/631 761	0.92	0.90–0.93		41 113/615 068	0.88	0.86–0.89
	**Model 3**									
		PE	Non-PE ^b^ (ref)							
			PE	5473/66 089	1.54	1.48–1.59		9767/87 360	1.36	1.33–1.40
		High demands/Low control	Low strain (ref)							
			High strain ^e^	11 645/203 151	1.18	1.16–1.21		17 821/251 842	1.01	0.99–1.03
	**Model 4**									
		PE	Non-PE ^b^ (ref)							
			PE	5473/66 089	1.50	1.45–1.56		9767/87 360	1.33	1.29–1.37
		Low demands/Low control	Active job ^f^ (ref)							
			Passive job	26 004/442 874	1.18	1.15–1.20		34 031/384 658	1.18	1.17–1.2
						
**Substance use disorders**										
	**Model 1**									
		PE	Non-PE ^b^ (ref)							
			PE	3161/66 089	1.81	1.69–1.94		1782/87 360	1.30	1.20–1.41
		Control	High control ^c^ (ref)							
			Low control	20 074/646 025	1.25	1.22–1.28		9 926/636 500	1.17	1.14–1.21
	**Model 2**									
		PE	Non-PE ^b^ (ref)							
			PE	3161/66 089	1.71	1.63–1.80		1782/87 360	1.33	1.26–1.41
		Demands	Low demands ^d^ (ref)							
			High demands	13 337/631 761	0.86	0.84–0.88		6 548/615 068	0.81	0.78–0.84
	**Model 3**									
		PE	Non-PE ^b^ (ref)							
			PE	3161/66 089	1.72	1.65–1.81		1782/87 360	1.37	1.30–1.44
		High demands/Low control	Low strain (ref)							
			High strain ^e^	5 835/203 151	1.11	1.08–1.15		2 699/251 842	0.93	0.89–0.97
	**Model 4**									
		PE	Non-PE ^b^ (ref)							
			PE	3161/66 089	1.81	1.72–1.91		1782/87 360	1.29	1.20–1.38
		Low demands/Low control	Active job ^f^ (ref)							
			Passive job	14 259/442 874	1.18	1.15–1.21		7 227/384 658	1.26	1.22–1.31
							
**Suicide attempt**										
	**Model 1**									
		PE	Non-PE ^b^ (ref)							
			PE	479/66 089	1.83	1.53–2.20		732/87 360	1.55	1.36–1.77
		Control	High control ^c^ (ref)							
			Low control	3095/64 6025	1.53	1.43–1.63		3 639/636 500	1.38	1.31–1.46
	**Model 2**									
		PE	Non-PE ^b^ (ref)							
			PE	479/66 089	1.58	1.39–1.78		732/87 360	1.38	1.26–1.51
		Demands	Low demands ^d^ (ref)							
			High demands	1658/631 761	0.78	0.73–0.84		1 901/615 068	0.69	0.65–0.74
	**Model 3**									
		PE	Non-PE ^b^ (ref)							
			PE	479/66 089	1.62	1.45–1.82		732/87 360	1.46	1.34–1.59
		High demands/Low control	Low strain (ref)							
			High strain ^e^	834/203 151	1.15	1.06–1.25		884/251 842	0.97	0.90–1.05
	**Model 4**									
		PE	Non-PE ^b^ (ref)							
			PE	479/66 089	1.75	1.53–1.99		732/87 360	1.46	1.30–1.64
		Low demands/Low control	Active job ^f^ (ref)							
			Passive job	2261/442 874	1.38	1.30–1.48		2775/384 658	1.46	1.38–1.54

^a^ OR adjusted for age, educational level, any mental disorder prior to 2006, parents’ depression diagnosis. ^b^ Non- PE (PE score<-3). ^c^ High control (>median job control score). ^d^ Low demands (>median job demands score). ^e^ High strain (high demands=0 and low control=0). ^f^ Active job (low control=0 and high demands=1).

Workers with PE compared to non-PE had increased odds of developing CMD, alcohol and substance use disorders, and suicide attempts, both among men and women. At the same time, workers with low compared to high control had increased odds of being diagnosed (model 1), while those with high compared to low demands had decreased odds (model 2), in both sexes. Workers with high strain had increased odds of developing CMD, alcohol and substance use disorders, and suicide attempts among men, while women had increased odds for CMD but decreased odds for alcohol and substance use disorders, and suicide attempts. Those with passive job had increased odds of being diagnosed with CMD, alcohol and substance use disorders, and suicide attempts.

### Mediation effects

Table 3 presents both the controlled direct and natural indirect effect of PE on CMD, alcohol and substance use disorders, and suicide attempt separately for men and women. The OR corresponding to natural indirect effects were very low, therefore the mediation effect of PRF can be considered practically null.

**Table 3:** Direct and indirect effects of precarious employment (PE) (in 2005) on common mental disorders, alcohol and substance use disorders, and suicide attempt (2006–2016) through low control, high demands, high strain, and passive job (in 2005).

			Men					Women		
			Cases/Exposed	OR ^a^	95% CI			Cases/Exposed	OR ^a^	95% CI
**Common mental disorders**										
	Model 1	Controlled direct effect (PE)	5473/ 66 089	1.45	1.39–1.50			9767/87 360	1.31	1.28–1.35
		Natural indirect effect (Low control)	37 649/646 025	1.02	1.01–1.03			51 852/636 500	1.00	1.00–1.01
	Model 2	Controlled direct effect (PE)	5473/66 089	1.49	1.43–1.56			9767/87 360	1.35	1.30–1.40
		Natural indirect effect (High demands)	27 921/631 761	1.00	0.99–1.00			41 113/615 068	1.01	1.01–1.02
	Model 3	Controlled direct effect (PE)	5473/66 089	1.31	1.24–1.38			9767/87 360	1.22	1.15–1.29
		Natural indirect effect (High strain)	11 645/203 151	1.00	0.99–1.01			17 821/251 842	1.00	1.00–1.00
	Model 4	Controlled direct effect (PE)	5473/66 089	1.52	1.45–1.59			9767/87 360	1.32	1.28–1.37
		Natural indirect effect (Passive job)	26 004/442 874	0.99	0.99–1.00			34 031/384 658	1.01	1.01–1.01
**Alcohol and substance use disorders**										
	Model 1	Controlled direct effect (PE)	3161/66 089	1.65	1.57–1.73			1782/87 360	1.37	1.28–1.46
		Natural indirect effect (Low control)	20 074/646 025	1.01	1.01–1.02			9 926/636 500	1.01	1.00–1.01
	Model 2	Controlled direct effect (PE)	3161/66 089	1.77	1.67–1.88			1782/87 360	1.31	1.19–1.44
		Natural indirect effect (High demands)	13 337/631 761	1.00	0.99–1.00			6 548/615 068	1.03	1.02–1.05
	Model 3	Controlled direct effect (PE)	3161/66 089	1.64	1.52–1.77			1782/87 360	1.21	1.04–1.40
		Natural indirect effect (High strain)	5 835/203 151	1.01	1.00–1.02			2 699/251 842	1.01	1.00–1.01
	Model 4	Controlled direct effect (PE)	3161/66 089	1.64	1.54–1.74			1782/87 360	1.38	1.28–1.48
		Natural indirect effect (Passive job)	14 259/442 874	1,00	0.99–1.00			7 227/384 658	1.02	1.01–1.03
**Suicide attempt**										
	Model 1	Controlled direct effect (PE)	479/66 089	1.46	1.30–1.63			732/87 360	1.33	1.20–1.47
		Natural indirect effect (Low control)	3095/64 6025	1.02	1.00–1.04			3639/636 500	1.01	1.00–1.01
	Model 2	Controlled direct effect (PE)	479/66 089	1.66	1.43–1.94			732/87 360	1.40	1.19–1.65
		Natural indirect effect (High demands)	1658/631 761	0.99	0.99–1.00			1901/615 068	1.05	1.03–1.08
	Model 3	Controlled direct effect (PE)	479/66 089	1.40	1.16–1.69			732/87 360	1.15	0.90–1.46
		Natural indirect effect (High strain)	834/20 3151	1.00	0.97–1.03			884/251 842	1.01	1.00–1.01
	Model 4	Controlled direct effect (PE)	479/66 089	1.48	1.28–1.71			732/87 360	1.33	1.20–1.49
		Natural indirect effect (Passive job)	2261/442 874	0.99	0.99–1.00			2775/384 658	1.02	1.01–1.03

^a^ OR adjusted for age, educational level, any mental disorder prior to 2006, parents’ depression diagnosis.

### Sensitivity analysis

After conducting sensitivity analyses, there were no relevant changes in the indirect effect trends observed in the main analysis, except that when applying the multilevel analysis, some natural indirect effects were higher. Notably, high demands (OR 1.21; 95% CI 1.16–1.27) and passive job (OR 1.10; 95% CI 1.08–1.13) for suicide attempts among women. These results suggest that using single level data with an aggregated mediator may slightly underestimate the mediating effects. However, the estimated OR for the natural indirect effects were still rather close to 1 in most cases, suggesting that psychosocial conditions do not have a mediating effect on the association between PE and mental health outcomes, as concluded from of the main analysis.

On the other hand, the association between PE and mental health outcomes tends to increase when not adjusted for previous psychiatric diagnoses and when adjusted for children among both sexes.

## Discussion

This is the first article analyzing the role of the psychosocial work environment as a possible pathway in the relationship between PE and poor mental health in the Swedish workforce, extending previous research on the negative impact of PE on mental health. Based on previous conceptual frameworks and cross-sectional studies, it was hypothesized that PRF mediate the association between PE and workers’ mental health. To test this hypothesis, the indirect effects of low control, high demand, high strain, and passive job were estimated. The results did not support the mediation hypothesis considering that the indirect effects were either low, null, or not significant, while the controlled direct effects showed a relatively strong influence of PE on mental health (although with differences in the magnitudes between the three outcomes). In other words, when estimating the effect of PE and PRF concomitantly in a mediation model, PE affected mental health directly without the mediation of PRF.

Despite the lack of mediating effect of PRF in the present study, results showed that – after adjusting for PRFs – PE increased the risk of CMD, substance use disorders, and suicide attempts in both sexes. Consistently, a previous study using the same data found associations between PE with all three outcomes (39). Thus, these results show that psychosocial conditions do not have a clear mediating effect on the association between PE and mental health when measured using the JEM.

Furthermore, differences between sexes were found in the association of PE and mental health outcomes. Despite women being more exposed to PE and mental disorders than men, the association was stronger among men. Very similar results were found in a recent study that showed a stronger association between PE and poor mental health among men, despite women being more exposed to both phenomena (40). Based on gender theory, as a hypothesis to explain this apparently contradictory result, it could be argued that the perception of the risk of PE could depend on the expectations of everyone based on their position in labor markets, which are strongly segregated by gender. Thus, while women could tend to normalize PE or non-standard employment since it allows them to balance work with family responsibilities, for men it could be less expected and consequently they tend to be more affected by it.

At the same time, differences between men and women were also found in the association of PE and PRF. Among men, PE increased the odds of low control and high demands. Consequently, the association with high strain was positive, while the one with passive job was negative. Among women, PE was positively associated with low control but negatively associated with high demands. These results suggest that the most precarious occupations have different psychosocial characteristics for men and women. While precarious jobs tend to be high strain for men, among women, they tend to be passive jobs. The main reason for this difference could be vertical and horizontal occupational gender segregation, a persistent problem in numerous labor markets, including the Swedish one (41, 42), which means that men have access to jobs with higher social and/or financial recognition and are provided with more responsibilities. In contrast, women have access mostly to jobs with less social and financial recognition, performing simpler tasks or tasks perceived as having less responsibility (34, 35). This asymmetry could imply differential exposures to psychological demands but also a gap in the individual’s ability to make decisions about his/her own job and to influence the work group or company policy.

Consistently with recent previous studies (16, 17, 44), it was found that low control was associated with an increased risk of CMD, substance use disorders, and suicide attempts for both sexes, although the association was higher among men. High demands was associated with a decreased risk of CMD, substance use disorders, and suicide attempts for both men and women.

### Limitations and strengths

A first limitation would be that multi-categorical measures of PE and mediators are more appropriate than dichotomous measures as they make it possible to identify different types and levels of precariousness and psychosocial exposure. Thus, the heterogeneity of the employment situations among the working population could be better captured with this approach. However, a binary variable was used to measure the exposure. To address this restriction, we performed sensitivity analyses, changing the dichotomization of the exposure and the mediators by defining alternative cut-off points. However, no significant differences were found.

Next, controlled direct effect’s estimation requires no unmeasured common causes possibly linking (i) the exposures and the outcomes and (ii) the mediators and the outcomes. In addition, for natural effects, there must be no unmeasured common causes of exposure and mediation. According to previous studies, given the likelihood of residual confounding for these exposures and mediators, the interpretation of the results emphasizes the direction rather than the precise magnitudes of estimated effects (37) The limitation of the mediation model used in this study to address a possible job change after 2005 could have caused unmeasured changes in demand/control potentially mediating a diagnosis of CMD, substance use disorders, or suicide attempts. However, a recent study based on the same cohort found a strong correlation between occupational titles from 2006 to 2016 (30–60 age range) (45). Therefore, it can be expected that the psychosocial exposure estimated in 2005 was consistent throughout the follow-up period. Finally, JEM reduce the bias that can occur when someone with mental health problems may rate their exposure level differently than those without mental health problems, but this leads to misclassification due to not accounting for differences within jobs. Complementarily, the study is partly based on register data and partly on a JEM, which both address the common method bias issue, where reports of exposure can be biased by health (46).

Despite these limitations, to the best of our knowledge, this is the first study analyzing the psychosocial work environment as a possible mediator in the relationship between multidimensional PE and register-based mental health indicators for a cohort of workers. Thus, this study advances current knowledge about the causal pathways that link PE and mental health, a recognized knowledge gap in previous epidemiological research (5, 9).

### Concluding remarks

The results of this study provide evidence for the pathways through which PE affects health. In the Swedish labor market, psychosocial conditions do not appear to have a mediating effect on the association between PE and CMD, substance use disorders and suicide attempts. Therefore, future studies in different contexts should further explore these findings.

## Supplementary material

Supplementary material
