# Supplementary material for: Can psychosocial risk factors mediate the association between precarious employment and mental health problems in Sweden? Results from a register-based study
Source: Scand J Work Environ Health. 2024 Apr 29;50(4):268–78. doi: 10.5271/sjweh.4151 (PMC11129838; doi:10.5271/sjweh.4151)
Supplement: Supplementary material [file SJWEH-50-268-S001.pdf]

Can psychosocial risk factors mediate the association between precarious employment and mental health problems in Sweden? Results from a register-based study[1]

by Fabrizio Méndez-Rivero, PhD,[2] Nuria Matilla-Santander, PhD, Virginia Gunn, PhD, David H Wegman, MD, Julio C Hernando-Rodriguez, PhD, Signild Kvart, MSc,, Mireia Julià, PhD, Bertina Kreshpaj, PhD, Theo Bodin, PhD, Tomas Hemmingsson, PhD, Carles Muntaner, PhD, Eva Padrosa, PhD, Melody Almroth, PhD

1. Supplementary material
2. Correspondence to: GREDS (Research Group on Health Inequalities, Environment, and Employment Conditions Network), Universitat Pompeu Fabra, 25-27 Ramon Trias Fargas Street, Ciutadella Campus, Mercè Rodoreda Building, 08005 Barcelona, Spain. [E-mail: fabrizio.mendez@upf.edu].

Table S1- Items used to assign level of control and demands Job Exposure Matrices (JEM)

**JEM Variables**	**Question**	**Answer Options**
Control	Can you partially decide when tasks should be done?	Never, Mostly not, Mostly, Always
	Do you have the opportunity to decide your own work pace?	Not at all, Occasionally, Roughly ¼ of the time, Half the time, Roughly ¾ of the time, Almost all the time
	Can you take short breaks to talk pretty much any time?	Not at all, Occasionally, roughly ¼ of the time, Half the time, Roughly ¾ of the time, Almost all the time
	Are you ever involved in deciding how your work is organized?	Never, Mostly not, Mostly, Always
Psychosocial demands	Are you sometimes so stressed that you do not have time to talk about or even think about something besides work?	Not at all, Occasionally, Roughly ¼ of the time, Half the time, Roughly ¾ of the time, Almost all the time
	Do you sometimes have so much to do that you have to work during lunch, work overtime, or take work home?	Not at all, A few days per month, One day per week, A few days per week, Every day
	Does your work require all of your attention and concentration?	Not at all, Occasionally, Roughly ¼ of the time, Half the time, Roughly ¾ of the time, Almost all the time

Supplementary Table S2- Direct and indirect effects of ‘Precarious employment’ (in 2005) on ‘Common mental disorders’, ‘Alcohol and substance use disorders’ and ‘Suicide attempt’ (from 2006 to 2016) through ‘Low control’, ‘High demands’, ‘High strain’ and ‘Passive job’ (in 2005), separately for men and women.

		Men				Women			
		N Case/ Exposed	Odds ratio	95%CI		N Case/ Exposed	Odds ratio	95%CI	
		**Common mental disorders**							
Model 1	Controlled direct effect (precarious employment)	10505/134932	1,42	1,38	1,46	19208/179977	1,33	1,30	1,36
	Natural indirect effect ***(Low control)***	37649/646025	*1,02*	*1,01*	*1,02*	51852/636500	*1,00*	*1,00*	*1,01*
Model 2	Controlled direct effect (precarious employment)	10505/134932	1,48	1,43	1,53	19208/179977	1,32	1,29	1,36
	Natural indirect effect ***(High demands)***	27921/631761	*1,00*	*1,00*	*1,00*	41113/615068	*1,02*	*1,01*	*1,02*
Model 3	Controlled direct effect (precarious employment)	10505/134932	1,29	1,23	1,35	19208/179977	1,24	1,19	1,30
	Natural indirect effect ***(High strain)***	11645/203151	*1,00*	*1,00*	*1,01*	17821/251842	*1,00*	*1,00*	*1,00*
Model 4	Controlled direct effect (precarious employment)	10505/134932	1,48	1,43	1,54	19208/179977	1,34	1,30	1,37
	Natural indirect effect ***(Passive job)***	26004/442874	*1,00*	*1,00*	*1,00*	34031/384658	*1,01*	*1,01*	*1,01*
		**Alcohol and substance use disorders**							
Model 1	Controlled direct effect (precarious employment)	5854/134932	1,56	1,50	1,62	3449/179977	1,34	1,27	1,41
	Natural indirect effect ***(Low control)***	20074/646025	*1,01*	*1,01*	*1,02*	9926/636500	*1,01*	*1,01*	*1,01*
Model 2	Controlled direct effect (precarious employment)	5854/134932	1,67	1,59	1,75	3449/179977	1,29	1,20	1,38
	Natural indirect effect ***(High demands)***	13337/631761	*1,00*	*1,00*	*1,00*	6548/615068	*1,03*	*1,02*	*1,04*
Model 3	Controlled direct effect (precarious employment)	5854/134932	1,58	1,48	1,68	3449/179977	1,23	1,11	1,37
	Natural indirect effect ***(High strain)***	5835/203151	*1,01*	*1,00*	*1,01*	2699/251842	*1,00*	*1,00*	*1,01*
Model 4	Controlled direct effect (precarious employment)	5854/134932	1,54	1,47	1,61	3449/179977	1,33	1,26	1,41
	Natural indirect effect ***(Passive job)***	14259/442874	*1,00*	*1,00*	*1,00*	7227/384658	*1,02*	*1,01*	*1,02*
		**Suicide attempt**							
Model 1	Controlled direct effect (precarious employment)	935/134932	1,53	1,40	1,67	1358/179977	1,27	1,17	1,37
	Natural indirect effect ***(Low control)***	3095/646025	*1,02*	*1,01*	*1,04*	3639/636500	*1,01*	*1,00*	*1,01*
Model 2	Controlled direct effect (precarious employment)	935/134932	1,70	1,51	1,92	1358/179977	1,32	1,17	1,50
	Natural indirect effect ***(High demands)***	1658/631761	*1,00*	*1,00*	*1,00*	1901/615068	*1,05*	*1,03*	*1,06*
Model 3	Controlled direct effect (precarious employment)	935/134932	1,43	1,23	1,67	1358/179977	1,17	0,97	1,39
	Natural indirect effect ***(High strain)***	834/203151	*1,00*	*0,98*	*1,01*	884/251842	*1,00*	*1,00*	*1,01*
Model 4	Controlled direct effect (precarious employment)	935/134932	1,58	1,42	1,75	1358/179977	1,26	1,15	1,38
	Natural indirect effect ***(Passive job)***	2261/442874	*1,00*	*0,99*	*1,00*	2775/384658	*1,02*	*1,01*	*1,03*

Odds ratio adjusted for age, educational level, any mental disorder prior to 2006, parents’ depression diagnosis.

Supplementary Table S3- Multilevel estimation of direct and indirect effects of 'Precarious employment' (in 2005) on 'Common mental disorders', 'Alcohol and substance use disorders' and 'Suicide attempt' (from 2006 to 2016) through 'Low control', 'High demands', 'High strain' and 'Passive job' (in 2005), separately for men and women.

		Men			Women		
		N Case/ Exposed	Odds ratio	95%CI	N Case/ Exposed	Odds ratio	95%CI
		**Common mental disorders**					
Model 1	Controlled direct effect (precarious employment)	32 483/525 931	1.35	1.32-1.38	65 824/813 199	1.15	1.13-1.16
	Natural indirect effect ***(Low control)***	37 649/646 025	*1.06*	*1.05-1.06*	51 852/636 500	*1.02*	*1.01-1.02*
Model 2	Controlled direct effect (precarious employment)	32 483/525 931	1.36	1.33-1.39	65 824/813 199	1.12	1.09-1.14
	Natural indirect effect ***(High demands)***	27 921/631 761	*1.01*	*1.00-1.01*	41 113/615 068	*1.06*	*1.05-1.07*
Model 3	Controlled direct effect (precarious employment)	32 483/525 931	1.10	1.05-1.15	65 824/813 199	0.99	0.96-1.02
	Natural indirect effect ***(High strain)***	11 645/203 151	*1.00*	*0.99-1.00*	17 821/251 842	*1.01*	*1.00-1.01*
Model 4	Controlled direct effect (precarious employment)	32 483/525 931	1.45	1.42-1.49	65 824/813 199	1.31	1.25-1.37
	Natural indirect effect ***(Passive job)***	26 004/442 874	*1.01*	*1.00-1.01*	34 031/384 658	*1.05*	*1.04-1.05*
		**Alcohol and substance use disorders**					
Model 1	Controlled direct effect (precarious employment)	17 928/525 931	1.33	1.29-1.38	13 305/813 199	1.34	1.27-1.41
	Natural indirect effect ***(Low control)***	20 074/646 025	*1.03*	*1.02-1.03*	9 926/636 500	*1.02*	*1.02-1.03*
Model 2	Controlled direct effect (precarious employment)	17 928/525 931	1.38	1.37-1.44	13 305/813 199	1.20	1.14-1.26
	Natural indirect effect ***(High demands)***	13 337/631 761	*1.01*	*1.01-1.02*	6 548/615 068	*1.09*	*1.06-1.12*
Model 3	Controlled direct effect (precarious employment)	17 928/525 931	1.13	1.06-1.21	13 305/813 199	1.09	1.01-1.17
	Natural indirect effect ***(High strain)***	5 815/203 151	*0.99*	*0.98-1.00*	2 699/251 842	*1.02*	*1.01-1.02*
Model 4	Controlled direct effect (precarious employment)	17 928/525 931	1.40	1.35-1.45	13 305/813 199	1.47	1.32-1.63
	Natural indirect effect ***(Passive job)***	14 259/442 874	*1.00*	*1.00-1.01*	7 227/384 658	*1.06*	*1.05-1.07*
		**Suicide attempt**					
Model 1	Controlled direct effect (precarious employment)	2 569/525 931	1.20	1.11-1.29	4 646/813 199	1.25	1.14-1.36
	Natural indirect effect ***(Low control)***	3 095/646 025	*1.06*	*1.04-1.09*	3 639/636 500	*1.04*	*1.03-1.06*
Model 2	Controlled direct effect (precarious employment)	2 569/525 931	1.41	1.28-1.56	4 646/813 199	1.13	1.02-1.23
	Natural indirect effect ***(High demands)***	1 658/631 761	*1.02*	*1.01-1.03*	1 901/615 068	*1.21*	*1.16-1.27*
Model 3	Controlled direct effect (precarious employment)	2 569/525 931	1.04	0.88-1.23	4 646/813 199	0.93	0.82-1.07
	Natural indirect effect ***(High strain)***	834/203 151	*1.00*	*0.98-1.01*	884/251 842	1.02	1.01-1.03
Model 4	Controlled direct effect (precarious employment)	2 569/525 931	1.26	1.17-1.37	4 646/813 199	1.27	1.08-1.49
	Natural indirect effect ***(Passive job)***	2 261/442 874	*1.01*	*1.00-1.01*	2 775/384 658	*1.10*	*1.08-1.13*
